# Detection and Characterization of Circulating Tumor Cells in Colorectal Cancer Patients via Epithelial–Mesenchymal Transition Markers

**DOI:** 10.3390/cancers17020303

**Published:** 2025-01-18

**Authors:** Yusuke Takahashi, Yuichi Ijiri, Shiki Fujino, Nakhaei Elnaz, Ayuko Kishimoto, Kentaro Shirai, Shigeki Iwanaga, Masatoshi Yanagida, Ali Asgar S. Bhagat, Norikatsu Miyoshi

**Affiliations:** 1 Department of Central Research Laboratories, Sysmex Corporation, Kobe 651-2271, Japan; takahashi.yusuke@sysmex.co.jp (Y.T.); ijiri.yuichi@sysmex.co.jp (Y.I.); nakhaei.elnaz@sysmex.co.jp (N.E.); kishimoto.ayuko@sysmex.co.jp (A.K.); shirai.kentaro@sysmex.co.jp (K.S.); iwanaga.shigeki@sysmex.co.jp (S.I.); yanagida.masatoshi@sysmex.co.jp (M.Y.); 2 Department of Gastroenterology, Central Clinical School, Monash University, Melbourne 3004, Australia; sfujino@gesurg.med.osaka-u.ac.jp; 3 Innovative Oncology Research and Regenerative Medicine, Osaka International Cancer Institute, Osaka 541-8567, Japan; 4 Department of Gastroenterological Surgery, Graduate School of Medicine, Osaka University, Suita 565-0871, Japan; 5 Biolidics Limited, Singapore 577177, Singapore; ali@nus.edu.sg; 6 Institute for Health Innovation and Technology (iHealthtech), National University of Singapore, Singapore 119276, Singapore; 7 Department of Biomedical Engineering, College of Design and Engineering, National University of Singapore, Singapore 117575, Singapore

**Keywords:** liquid biopsy, circulating tumor cells, colorectal cancer, adenomatous polyposis coli

## Abstract

**Background/Objectives:** Liquid biopsy methods have gained prominence as minimally invasive tools to improve cancer treatment outcomes. Circulating tumor cells (CTCs) offer valuable insights into both primary and metastatic lesions. However, validating the CTC test results requires confirmation that the detected cells originate from cancer tissue. While studies have identified CTCs in colorectal cancer (CRC) patients using molecular markers, simultaneous validation of their cancer tissue origin remains unexplored. **Methods:** This study introduces a simple approach to detect adenomatous polyposis coli (APC) gene abnormalities alongside established CTC markers using a molecular imaging flow cytometer (MI-FCM). Given that APC gene abnormalities occur in 60–70% of CRC patients, their detection serves as strong evidence of cancer origin. **Results:** Our method achieved 92% concordance with DNA sequence analysis of tumor-derived cells. In a proof-of-concept study using 5 mL of whole blood from CRC patients, we observed a high frequency of cells exhibiting APC abnormalities, cytokeratin (CK), and vimentin (Vim) expression. Extending the study to 80 CRC patients across pathological stages I–IV confirmed CK and Vim as valid CTC markers. Three distinct cell populations were identified in blood: CK+/Vim−, CK+/Vim+, and CK−/Vim+. CTC number and frequency increased progressively with cancer stage. **Conclusions:** This is the first report demonstrating CK and Vim as effective markers for direct CTC detection in CRC patients. Our findings provide evidence-based validation of CTC markers, offering new insights and advancing approaches for patient care.

## Simple Summary

Circulating tumor cells (CTCs) are promising targets for liquid biopsy, providing valuable information for selecting appropriate treatments. Ensuring the reliability of CTC test results requires verifying the cancerous origin of detected cells. However, a method to simultaneously validate traditional CTC markers and the cancerous origin has not been established. This study developed a method using a molecular imaging flow cytometer (MI-FCM) to detect abnormal adenomatous polyposis coli (APC) genes along with established CTC markers. A proof-of-concept using whole blood from colorectal cancer patients showed high concordance between CTC markers and cells with APC abnormalities. Additionally, the number and frequency of CTCs increased in a pathology stage-dependent manner. This is the first report demonstrating that traditional CTC markers are effective for direct CTC detection in colorectal cancer patients. This evidence-based assessment of CTC markers is expected to advance medical approaches, providing valuable insights for patient care.

## 1. Introduction

Circulating tumor cells (CTCs) are solid cancer-derived cells that travel through the bloodstream [1,2,3]. Extensive research has investigated the link between CTCs and metastasis across various cancer types. The presence of CTCs elevates metastasis risk and reduces the overall survival rates [4,5,6,7]. Additionally, CTCs have gained attention as targets for liquid biopsies, offering noninvasive insights into cancer cell properties through blood samples as an alternative to invasive biopsies [8,9,10,11].

Accurate CTC testing requires confirming that the detected cells originate from cancer tissue. Traditionally, cytokeratin (CK) and Vim have been common markers for CTC detection [12,13]. Although some studies have identified CK- and/or vimentin (Vim)-expressing cells in blood as cancer-derived using DNA or RNA sequencing, the complexity of these methods limits their clinical utility [14,15,16]. Therefore, a streamlined approach is needed to detect CTCs using CK and/or Vim while verifying their cancerous origin.

In this study, we focused on utilizing the C-terminal truncated adenomatous polyposis coli (APC) protein as a marker for genetic mutation, a trait present in 60–70% of colorectal cancer (CRC) patients. This marker was used to confirm that the detected cells originated from the cancer tissue [17,18]. To date, no studies have successfully identified cancer-specific gene abnormalities within CTCs along with CK and/or Vim in CRC. Therefore, we developed a novel method to simultaneously detect mutant APC, CK, and/or Vim using fluorescence-labeled antibodies targeting both the N- and C-termini of APC. To evaluate the effectiveness of this approach, we analyzed isolated tumor-derived cancer cells (iCCs) obtained from surgically resected cancer tissues [19].The analytical performance of our method showed a 92% concordance rate with DNA sequence analysis in detecting APC abnormalities. Moreover, in iCCs with APC mutations, we observed three distinct CK and Vim expression patterns: CK+/Vim−, CK+/Vim+, and CK−/Vim+.

To validate our approach, we analyzed 5 mL of whole blood from CRC patients using a CTC detection system. This system combined a size-based CTC enrichment method utilizing dean flow fractionation, which prevents hemolysis, with imaging flow cytometry for precise detection of rare CTCs [20,21]. Performance evaluation with spike-recovery tests on various cell lines demonstrated ~50% recovery, independent of epithelial cell adhesion molecule (EpCAM) expression [20,21]. Additionally, it has been confirmed that no CTCs were detected in the whole blood of 38 healthy donors [21]. In patients with confirmed APC abnormalities in iCCs, 93% of APC-mutant cells in the blood were positive for CK and/or Vim. Conversely, among CK- and/or Vim-positive cells in the blood, 90% also exhibited APC abnormalities.

We expanded our analysis to include 80 patients with CRC across pathological stages I–IV. For this, we utilized CK and Vim as dependable CTC markers, as demonstrated in this study. The same three distinct cell populations identified during the iCC examination were observed: CK+/Vim−, CK+/Vim+, and CK−/Vim+. Both the quantity of CTCs and the proportion of patients with at least one CTC with the advancement of the pathological stages.

## 2. Materials and Methods

### 2.1. Cell Lines

Colon cancer cell lines HCT116 and DLD1, along with non-small-cell lung cancer lines A549 and HCC827, were acquired from the American Type Culture Collection (Manassas, VA, USA). The cells were cultured according to the manufacturer’s instructions. Afterward, the cancer cell lines were harvested using 0.25% trypsin/ethylene diaminetetraacetic acid (EDTA; Thermo Fisher Scientific, Waltham, MA, USA), suspended in CELLBANKER1 (ZENOAQ, Fukushima, Japan), and stored at −80 °C. Before initiating the experiments, the stored cell lines were thawed.

### 2.2. Isolated Tumor-Derived Cancer Cells (iCCs)

Tumor-derived cancer cells were established following the same protocol described previously [19]. These cells were isolated from surgically resected organ remnants of 13 CRC patients at the Osaka International Cancer Institute and Minoh City Hospital (Osaka, Japan). Sample collection spanned from June 2020 to May 2022.

### 2.3. Induction of Epithelial–Mesenchymal Transition

To generate a cell line positive for CK and/or Vim expression, HCC827 cells were cultured with 5 ng/mL of transforming growth factor-β (TGF-β) (PeproTech, Rocky Hill, CT, USA) for one month. The HCC827 cells undergoing epithelial–mesenchymal transition (EMT) induction were preserved following the same protocol used for cell line stock preparation.

### 2.4. Western Blotting

Cell lysates were prepared, and western blotting was conducted following previously described protocols [22]. The antibodies utilized included the APC-N antibody (Clone Ali 12-28; Santa Cruz Biotechnology, Dallas, TX, USA) and horseradish peroxidase-labeled anti-mouse IgG secondary antibody (Polyclonal, MBL, Tokyo, Japan). The chemiluminescent signals developed on the polyvinyledene fluoride (PVDF) membrane were captured using the ImageQuant^TM^ LAS 3000 imaging analyzer (Fujifilm, Tokyo, Japan). Precision Plus Protein Dual Color Standard was used as molecular weight markers (Bio-Rad, Hercules, CA, USA).

### 2.5. APC Gene Sequence

Genomic DNA extraction from the primary cells was carried out using a DNA extraction kit (Takara Bio, Kusatsu, Japan) according to the manufacturer’s protocol. PCR amplification employed the Hot Start High-Fidelity 2X Master Mix (New England Biolabs, Ipswich, UK) in accordance with the provided guidelines. The primer sequences were designed using the “Primer3” online tool accessed on 23 March 2020 (https://primer3.ut.ee/) (Table 1). Thermal cycling conditions included 15 s at 98 °C, followed by 15 s at 58 °C (Exon 13) or 62 °C (Exons 14 and 15), and 20 s at 72 °C (Exons 13 and 14) or 60 s at 72 °C (Exon 15). The process began with an initial 1 min at 98 °C and concluded with a 2 min final extension at 72 °C.

PCR products were separated via agarose gel electrophoresis, and the target bands were purified using the MinElute PCR Purification Kit (QIAGEN, Hilden, Germany) per the manufacturer’s instructions. Sequencing services were provided by Eurofins Genomics Co., Ltd. (https://eurofinsgenomics.jp/jp/home/). The nucleotide sequences were determined using the Sanger sequencing method, and the sequence information was obtained with an ABI3730XL (Thermo Fisher Scientific, Waltham, MA, USA). The resulting sequences were compared with the wild-type APC (*wAPC*) gene sequence (ACCESSION: NG_008481.4) using the basic local alignment search tool to detect and analyze potential mutations.

### 2.6. CTC Detection

Blood samples were obtained from 80 CRC patients treated at the Osaka International Cancer Institute and Minoh City Hospital (Osaka, Japan) between June 2020 and May 2022. Each sample was collected in EDTA blood collection tubes, with 10 mL drawn per donor. CTCs were isolated and enriched using a hybrid double-spiral microfluidic chip, following the protocol described previously [21]. After recovery from the chips, samples were fixed with 2% paraformaldehyde in phosphate-buffered saline and permeabilized using 90% methanol. The permeabilized samples were immunostained with the anti-APC-C antibody (Clone APC-C 28.9; Merck, Darmstadt, Germany) as the primary antibody. For secondary staining, Alexa-647 anti-mouse IgG antibody (Abcam, Bristol, UK) was applied. Additional coimmunostaining was performed using Alexa-488 anti-APC-N antibody (Clone Ali 12-28; Santacruz, Dallas, TX, USA), PE-Vimentin antibody (Clone V9; Santacruz, Dallas, TX, USA), e-fluoro615 anti-pan-cytokeratin antibody (Clone AE1/AE3; Thermo Fisher Scientific, Waltham, MA, USA), BV785 anti-CD16 antibody (Clone 3G8; BioLegend, San Diego, CA, USA), BV785 anti-CD34 antibody (Clone 561; BioLegend), and BV785 anti-CD45 antibody (Clone HI30; BioLegend). Hoechst 33342 (Dojindo, Kumamoto, Japan) was used for nuclear staining. Finally, the samples were analyzed using the molecular imaging flow cytometer (MI-FCM) system.

### 2.7. Characterization of the iCCs and CTCs

CD16, CD34, and CD45 were used as markers to identify white blood cells and distinguish them from CTCs or iCCs in the MI-FCM measurements. A fluorescence intensity threshold of 3000 was predefined, and cells with fluorescence intensities below this threshold were identified as potential iCC or CTC candidates. In MI-FCM analysis, APC parameters were employed to assess C-terminal deletion mutations in the APC gene. These parameters were calculated by dividing the fluorescence intensity of the antibody targeting the N-terminus of APC by that of the antibody targeting the C-terminus. An empirical threshold was established to detect C-terminal deletions, set such that 95% of iCCs with defective APC mutations fell below this threshold. The characterization of iCCs and CTCs through MI-FCM was based on the fluorescence intensities of CK and Vim, with 3000 set as the empirical threshold for marker positivity. The CTC count refers to the sum of CK+/Vim− cells, which are epithelial cell marker positive CTCs, CK−/Vim+ cells, which are mesenchymal cell marker positive CTCs, and CK+/Vim+ cells, which are intermediate cell marker positive CTCs between epithelial and mesenchymal. Additionally, the number of circulating tumor microemboli (CTMs) was evaluated as the number of aggregates of two or more homogeneous or heterogeneous CTCs, confirmed by visually inspecting the brightfield and HOECHST images of CTCs obtained with MI-FCM. The total number of detected CTCs and mesenchymal CTCs in each stage were compared using the Mann–Whitney U test.

### 2.8. Pathological Information of CRC Patients

The pathological stage information was obtained from the Osaka International Cancer Institute and Minoh City Hospital. The pathological stage of colorectal cancer was determined based on the combination of three factors: the depth of tumor invasion (T factor), lymph node metastasis (N factor), and distant metastasis (M factor).

## 3. Results

### 3.1. Study Concept

The primary objective of this study was to evaluate the reliability of CK and Vim as CTC markers in comparison with cancer-specific markers. To achieve this, we focused on a cancer-specific marker: the C-terminal truncated mutation of the APC protein, which is reported in 60–70% of CRC patients. Our approach involved three sequential steps to substantiate this concept.

First, we developed a method using MI-FCM to simultaneously detect mutant APC, CK, and Vim. The analytical performance of this method was assessed by comparing its concordance with APC gene abnormalities identified in iCCs through DNA sequencing. Additionally, we validated the method’s ability to detect APC abnormalities in conjunction with CK and/or Vim within iCCs.

**Figure 1:**
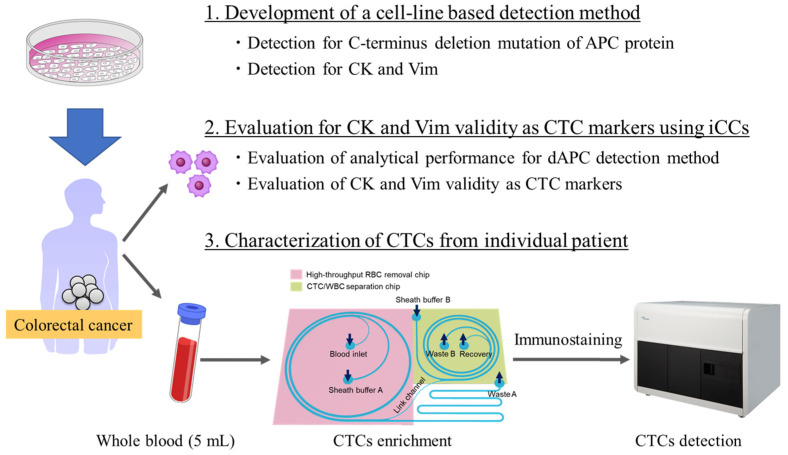
Study design. The study followed these stages sequentially: (1) development of the detection method using cell lines, (2) validation of Cytokeratin (CK) and Vimentin (Vim) as circulating tumor cell (CTC) markers using isolated tumor-derived cancer cells (iCCs), and (3) characterization of CTCs from individual patients.

Second, we evaluated the validity of CK and Vim as CTC markers by comparing the frequency of APC-mutant cells among CK- and/or Vim-positive cells in blood samples and the frequency of CK- and Vim-positive cells within APC-mutant cell populations. This step involved analyzing 5 mL of blood samples from CRC patients using our CTC detection system. Lastly, we identified CTCs using CK and Vim markers across a cohort of 80 CRC patients, covering pathological stages I–IV. This step aimed to clarify the relationship between CTC characteristics and pathological stages (Figure 1).

### 3.2. Construction of a Panel for the Detection of APC Mutation, CK, and Vim

**Figure 2:**
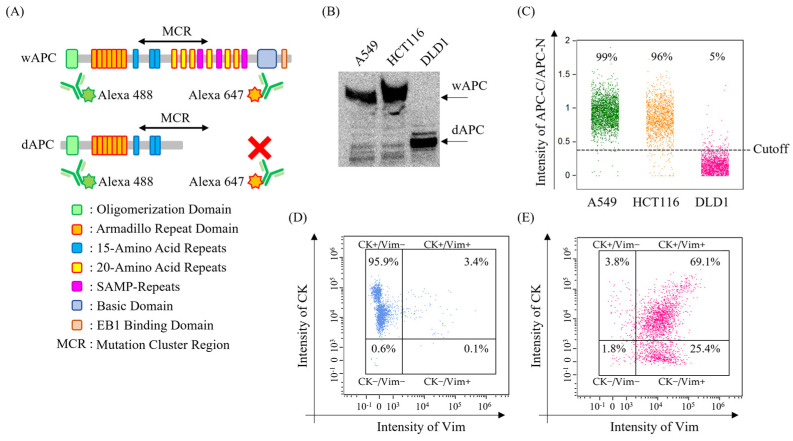
Development of APC mutation, CK, and Vim detection methods. (**A**) Image of the mutated APC detection method. (**B**) Western blot image of wild-type APC and mutant APC using A549, HCT116, and DLD1 cell lines. (**C**) Intensity of the APC-C/APC-N signal. Each cell line was immunostained and measured using imaging flow cytometer (MI-FCM). The cutoff was determined to encompass 95% of DLD1 cells exhibiting confirmed detection of mutant APC (dAPC) expression through Western blotting. (**D**,**E**) Expression of CK and Vim in HCC827 and transforming growth factor-beta (TGF-β)-stimulated HCC827. Each cell line was immunostained and measured using MI-FCM. The uncropped blots are shown in Figure S3.

A method was developed to detect the cancer-specific C-terminal deletion mutant of the APC protein (dAPC) using an Alexa488-labeled anti-APC antibody for the N-terminal and an Alexa647-labeled anti-APC antibody for the C-terminal (Figure 2A). In this setup, wAPC produces signals from both terminals, whereas dAPC shows a signal only from the N-terminal. This was confirmed using wAPC-positive and dAPC-positive cell lines. To verify APC’s molecular size, western blotting identified a 310 kDa full-length band in A549 and HCT116, with a lower molecular weight APC detected in DLD1 (Figure 2B). Immunostaining and MI-FCM analysis showed that the APC-C/APC-N intensity ratio exceeded the DLD1-based threshold in 99% of A549 and 96% of HCT116 cells (Figure 2C).

Next, HCC827 and TGF-β-stimulated HCC827 cell lines were used to evaluate CK and Vim as CTC markers. Coimmunostaining with anti-CK and anti-Vim antibodies was performed, followed by MI-FCM analysis. In HCC827 cells, the CK-positive rate was 95.9% (Figure 2D). However, in TGF-β-stimulated HCC827 cells, CK positivity dropped to 3.8%, while Vim positivity increased from 0.08% to 25.4%, and the double-positive rate for CK and Vim rose from 3.41% to 69.1% (Figure 2E). These results confirmed MI-FCM’s ability to simultaneously detect dAPC, CK, and Vim.

### 3.3. Evaluation of the Analytical Performance of the Constructed Method Using iCCs

**Figure 3:**
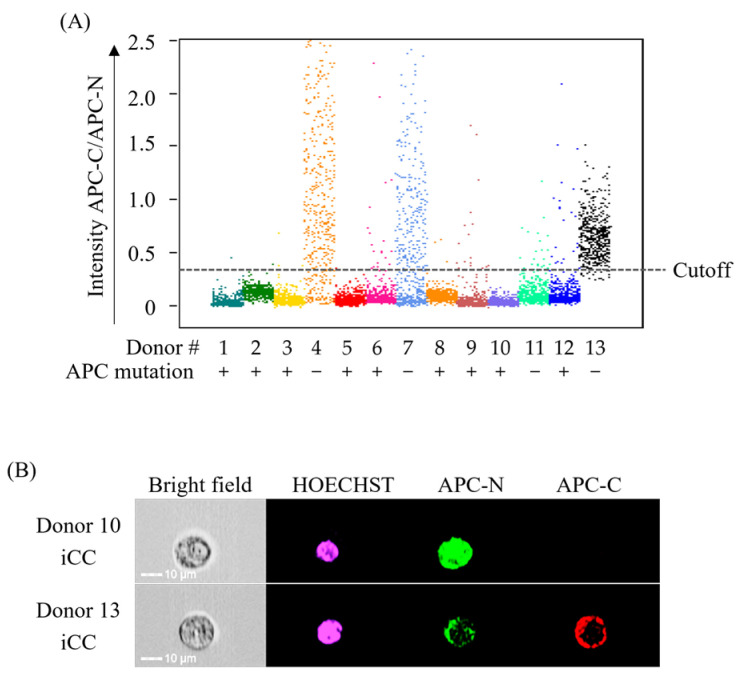
Validity assessment of the detection method for APC mutation using iCCs. (**A**) Intensity of APC-C/APC-N signals in iCCs obtained from 13 CRC patients. iCCs were immunostained and analyzed using MI-FCM. The sequencing results of the APC gene are listed alongside the corresponding donor numbers. (**B**) Immunostaining images of APC-mutated and APC-wild-type iCCs. APC-N (green) and APC-C (red) were detected using specific antibodies, and nuclei were counterstained with Hoechst 33342 (violet).

Compared to cancer cell lines, iCCs more closely mimic the characteristics of live cancer tissue. To validate the efficacy of the dAPC detection method developed with cancer cell lines, we assessed its performance using iCCs from 13 patients. First, gene sequencing of APC confirmed mutations in 9 of the 13 patients. Next, we evaluated the new CTC detection system, using APC mutation status as a benchmark. Applying a predefined threshold that captured 95% of APC-mutated iCCs, the sensitivity and specificity for detecting mutant APC were 100% and 75%, respectively (Table 2; Table S1). In the three cases with wild-type APC, the proportion of cells exceeding the threshold was 86%, 86%, and 95% (Figure 3A). Imaging showed that iCCs expressing wAPC stained for both the N- and C-terminus of APC, while those expressing dAPC stained only for the N-terminus (Figure 3B).

### 3.4. Validity Evaluation of CK and Vim as CTC Markers

**Figure 4:**
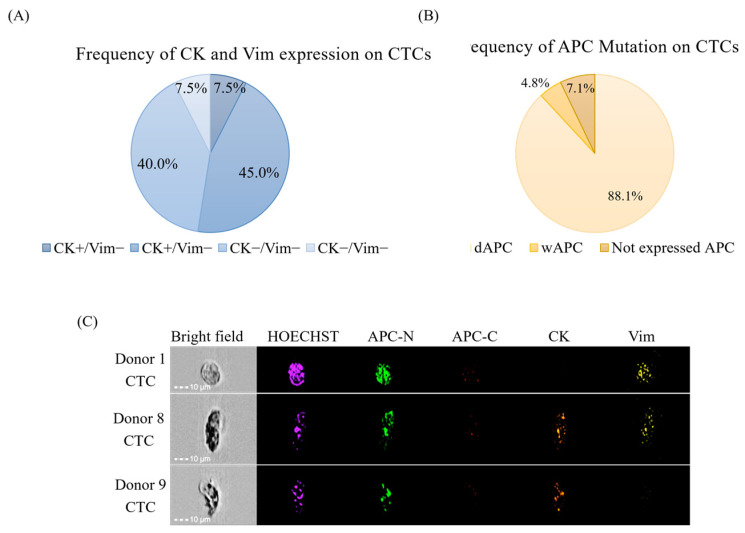
Evaluation of CK and Vim as CTC markers. Whole blood samples from nine patients with APC gene mutations detected in their iCCs were analyzed using the CTC detection system. (**A**) Frequency of APC mutations in CTCs. (**B**) Frequency of CK and Vim expression in CTCs. (**C**) Immunostaining image of APC-mutated CTCs, with APC-N (N-terminal of APC, green), APC-C (C-terminal of APC, red), CK (orange), and Vim (yellow) stained using specific antibodies. Nuclei were counterstained with Hoechst 33342 (violet).

To validate CK and Vim as reliable markers for CTC detection, we used our CTC detection system to identify dAPC-positive cells from the whole blood of nine patients with APC gene mutations in their iCCs. Across these patients, 40 dAPC-positive cells were identified. CK-positive cells constituted 8%, while 45% of the cells were positive for both CK and Vim, and 40% were Vim-positive (Figure 4A). Only 8% of the detected cells were negative for both CK and Vim (Figure 4A). This result indicates that CK and Vim have sufficient sensitivity as CTC markers.

We further analyzed APC expression within CK- and/or Vim-positive cells using the same dataset. Among these cells, 88% exhibited positive dAPC expression, whereas 7% lacked APC expression (Figure 4B,C). The findings revealed a strong concordance between the CK- or Vim-positive rates of dAPC-positive CTCs and the dAPC-positive rates within CK- or Vim-positive CTCs. This result indicates that CK and Vim are specific markers for cancer cells.

Collectively, these results confirm the validity of CK and Vim as effective CTC markers.

### 3.5. Detection of CTCs Using CK and Vim in CRC Patients

**Figure 5:**
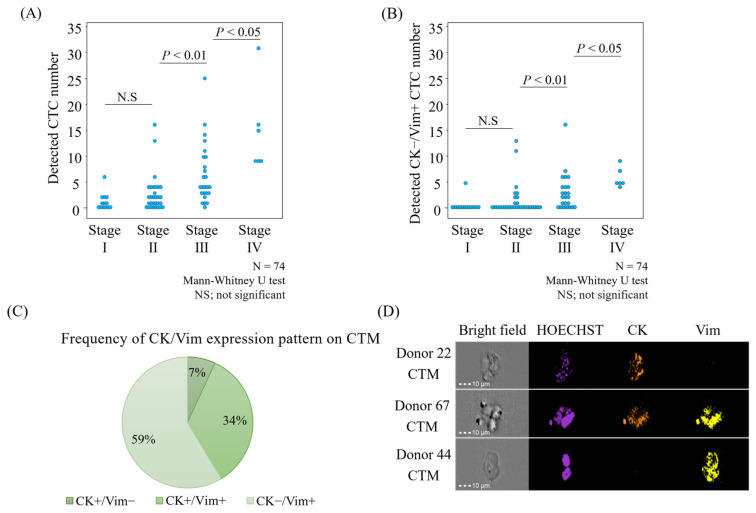
Analysis of CTCs from 74 patients with CRC. A 5 mL whole blood sample from each of the 74 patients with CRC was analyzed using the CTC detection system. Statistical comparisons were performed using the Mann–Whitney U-test. (**A**) Total number of CTCs expressing CK+/Vim−, CK+/Vim+, and CK−/Vim+ CTCs markers, grouped by pathological stage. (**B**) Number of CK−/Vim+ CTCs by pathological stage. (**C**) Frequency of CK and Vim expression in circulating tumor microemboli (CTMs). (**D**) Immunostaining image of CTMs, showing CK (orange) and Vim (yellow) staining with specific antibodies, and nuclei stained with Hoechst 33342 (violet).

After validating CK and Vim as effective CTC markers in CRC, we analyzed CTCs from the whole blood of 80 CRC patients. Six errors were identified, reducing the total number of valid cases to 74. The errors included three samples with liquid leakage during enrichment, one with poor immunostaining, one with coagulation preventing separation, and one with a corrupted MI-FCM data file. The distribution across the pathological stages included 13 cases in Stage I, 30 in Stage II, 25 in Stage III, and 6 in Stage IV (Table 3). The frequency of patients with at least one detected CTC was 62%, 73%, 96%, and 100% in Stages I, II, III, and IV, respectively (Table 3; Figure 5A). Additionally, the frequency of patients with at least one CK−/Vim+ CTC, which is characterized as a highly malignant mesenchymal cell, was 8%, 30%, 68%, and 100% in Stages I, II, III, and IV, respectively (Table 3; Figure 5B).

The incidence of circulating tumor microemboli (CTMs), defined as aggregates of two or more cells, increased with stage: 8%, 13%, 40%, and 83% in stages I, II, III, and IV, respectively. Analysis of molecular expression within CTMs showed 7% CK+/Vim−, 34% CK−/Vim+, and 59% CK+/Vim+, indicating a high proportion with mesenchymal properties (Table 3; Figure 5C,D). No CTCs were detected in four samples from healthy individuals (Figure S1).

## 4. Discussion

The role of CTCs in metastasis and prognosis is well recognized. While histopathological tissue analysis remains the gold standard for understanding cancer characteristics, its frequent application is limited by its invasive nature. Additionally, obtaining samples from small metastatic sites poses challenges for pathological evaluation. In contrast, molecular characterization of CTCs in blood offers a minimally invasive approach, enabling direct evaluation of cancer cell features associated with metastasis and prognosis.

Many studies have assessed CTCs using molecular markers such as CK and Vim. However, direct confirmation that the detected cells originate from cancer tissue has been insufficient. This study targeted C-terminal truncated mutations of the APC protein, observed in 60–70% of CRC patients. We developed a simple method to detect these dAPC mutations through immunostaining. Sequencing experiments using iCCs identified APC mutations in 9 of 13 cases, yielding a mutation frequency of 69%, consistent with previous reports [23]. The method’s analytical performance showed 92% concordance with DNA sequencing in detecting APC abnormalities. Some discrepancies arose, with sequencing identifying wild-type APC while immunostaining indicated mutations. These differences may reflect the sequencing scope, which covered exons 13–15—encompassing about 70–80% of known CRC mutation sites [24]. In one mismatched case, the mutation might have occurred on the N-terminal side of exon 12. Additionally, CTC analysis confirmed that CTCs from this patient exhibited dAPC mutation positivity.

In this study, we used iCCs to validate CK and Vim expression in cancer cells. Among patients with confirmed APC mutations in iCCs, 10% of dAPC-positive CTCs showed no CK or Vim expression. Notably, even in the immunostaining of iCCs, a small number of CK- and Vim-negative cells were detected, supporting the presence of such cells in blood (Figure S2). We also analyzed APC expression in CK- or Vim-positive blood cells from patients with APC abnormalities in their iCCs. Among these cells, 88% were dAPC-positive, while 5% were wAPC-positive. An empirical threshold was set to include 95% of iCCs with APC abnormalities, accounting for the observed 5% wAPC-positive ratio.

Upon detecting CTCs in whole blood from Stage I–IV CRC patients, both CTC counts and the frequency of patients with at least one detected CTC increased with advancing stage, aligning with prior clinical findings [25]. This study confirms CK and Vim as reliable CTC markers and offers compelling data on their clinical relevance. The proportion of patients with three or more mesenchymal CTCs approximately corresponds with the reported postoperative recurrence rates [26]. Similarly, CTM positivity per pathological stage aligns with these recurrence rates. Additionally, studies report elevated programmed death ligand 1 (PD-L1) levels in mesenchymal cells, suggesting that evaluating EMT in CTCs may inform the use of immune checkpoint inhibitors [27,28,29]. Recent studies on EMT have revealed that partial EMT cells, which exhibit characteristics of both epithelial and mesenchymal cells, contribute to the formation of CTC clusters, increased resistance to apoptosis, and the acquisition of treatment resistance [30]. In this study, CTC and CTM with partial EMT characteristics were also detected. By comparing the recurrence information of the patients measured in this study with the characteristics of CTC and CTM, including partial EMT, it may be possible to stratify patients who should be actively treated. Therefore, it is important to follow up on the prognosis of the patients registered in this study.

## 5. Conclusions

CK and Vim have traditionally been used as gold standard markers for CTCs. However, since CK and Vim are also expressed in normal cells, it has been unclear whether CK- and Vim-positive cells in the blood truly originate from cancer tissue. In this study, we demonstrated the validity of CK and Vim as CTC markers by simultaneously detecting the C-terminal deletion of the APC protein, which is derived from genetic mutations, along with CK and Vim. The measurement of blood from patients with confirmed APC gene mutations in primary cells showed that CK- and Vim-positive cells and dAPC-positive cells matched approximately 90%, indicating that CK and Vim are useful markers for CTCs.

Regardless of the APC gene mutation status, the results of CTC measurement using CK and Vim in all colorectal cancer patients also highlighted a stage-dependent increase in CTC number and frequency, with median CTC counts of 1, 2, 4, and 12 cells for Stages I, II, III, and IV, respectively. The frequency of patients with at least one detected CTC was 54%, 73%, 96%, and 100% in Stages I, II, III, and IV, respectively. To validate the clinical significance of CTC detection using CK and Vim in CRC, follow-up studies on patient outcomes and a detailed analysis of the relationship between CTCs and prognosis should be essential.

## Figures and Tables

**Table 1:** Primer list for the adenomatous polyposis coli (APC) sequence.

Primer Name	Sequence (5′–3′)
APC Exon13 Fw	TTTCTATTCTTACTGCTAGCATT
APC Exon13 Rev	ATACACAGGTAAGAAATTAGGA
APC Exon14 Fw	AGGGACGGGCAATAGGATAG
APC Exon14 Rev	GGTCTTTTTGAGAGTATGAATTCTG
APC Exon15-1 Fw	ACTGCATACACATTGTGACCT
APC Exon15-1 Rev	TCCCCGTGACCTGTATGGAG
APC Exon15-2 Fw	ACACCTCAAGTTCCAACCACA
APC Exon15-2 Rev	TCTGCCTCTTTCTCTTGGTTT
APC Exon15-3 Fw	GTTCATCCAGCCTGAGTGCT
APC Exon15-3 Rev	CAGGGGGCTCAGTCTCTTTG
APC Exon15-4 Fw	ACTCCGGTTTGCTTTTCTCA
APC Exon15-4 Rev	TCTTAAGGTTGGGCTTGGAGC
APC Exon15-5 Fw	GGACTAAATCAGATGAATAATGG
APC Exon15-5 Rev	CCATCAAGAGTGCCTCCCAA

**Table 2:** Performance of the detection of dAPC detection method.

		iCCs Gene Sequence	
		Mutant	Wild-Type
iCCs MI-FCM	Mutant	9	1
	Wild-type	0	3

**Table 3:** Number of detected CTCs and CTC-positive rate by stage in 74 patients.

Pathological Stage	No. of Patients	No. of PatientsCTC ≥ 1	CTC-Positive Rate	No. of Patients CK−/Vim+ CTC ≥ 1	CK−/Vim+ CTC-Positive Rate	No. of Patients CTM ≥ 1	CTM Positive Rate
Stage Ⅰ	13	8	62%	1	8%	1	8%
Stage Ⅱ	30	22	73%	10	33%	4	13%
Stage Ⅲ	25	24	96%	17	68%	9	36%
Stage Ⅳ	6	6	100%	6	100%	5	83%

## Data Availability

Data are available from the first author upon a reasonable request.

## Supplementary Materials

The following supporting information can be downloaded at: https://www.mdpi.com/article/10.3390/cancers17020303/s1. Figure S1: Detection of CTCs in healthy samples. Peripheral blood mononuclear cells (PBMCs) from four healthy donors were analyzed using the CTC detection system. (A) Total number of detected CTCs. (B) Immunostaining image of PBMCs. CK (orange), Vim (yellow), and CD16/34/45 (pink) were labeled with specific antibodies. Nuclei were counterstained with Hoechst 33342 (violet); Figure S2. CK and Vim expression patterns in iCCs. iCCs harboring an APC gene mutation were immunostained and analyzed using MI-FCM. (A) CK−/Vim+ iCCs. (B) CK+/Vim+ iCCs. (C) CK+/Vim− and CK−/Vim+ heterogeneous iCCs. (D) CK+/Vim− iCCs. Figure S3. Original Western blot image of Figure 2B. From left to right, the lanes show molecular weight markers, A549, HCT116, DLD1, LoVo, molecular weight markers, A549, HCT116, DLD1, and LoVo. The lysates of each cell line on the left side were stained with the anti-APC antibody clone F-13, and the lysates of each cell line on the right side were stained with the anti-APC antibody clone Ali 12-28. Table S1. The nucleotide sequences were determined using the Sanger sequencing method, and the sequence information was obtained with an ABI3730XL.
