# Supplementary figures and images for: Detection and Characterization of Circulating Tumor Cells in Colorectal Cancer Patients via Epithelial–Mesenchymal Transition Markers
Source: Cancers (Basel). 2025 Jan 18;17(2):303. doi: 10.3390/cancers17020303 (PMC11763958; doi:10.3390/cancers17020303)

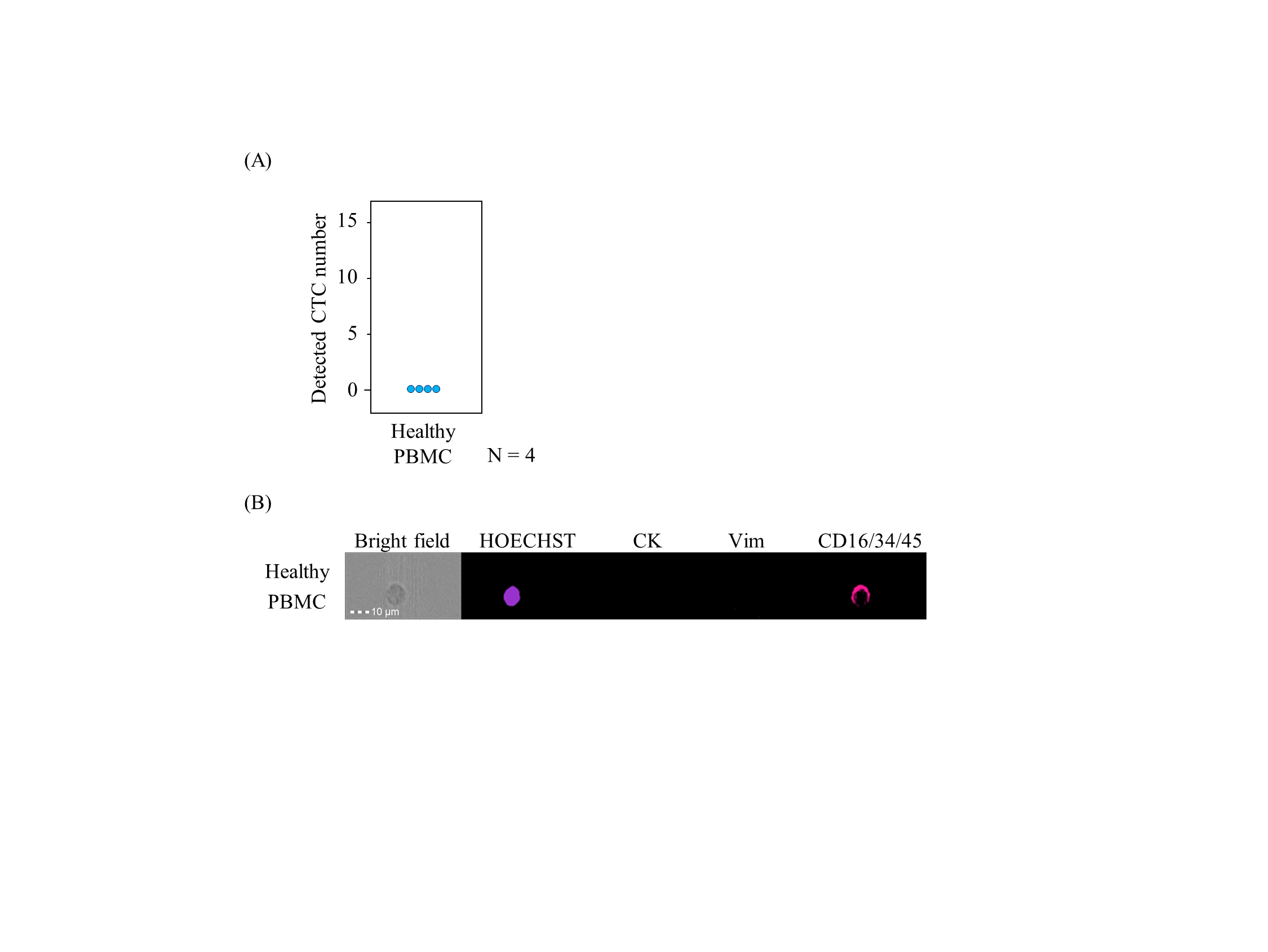

Supplement: Supplementary file 1 [file cancers-17-00303-s001.zip › Figure S1.tif]

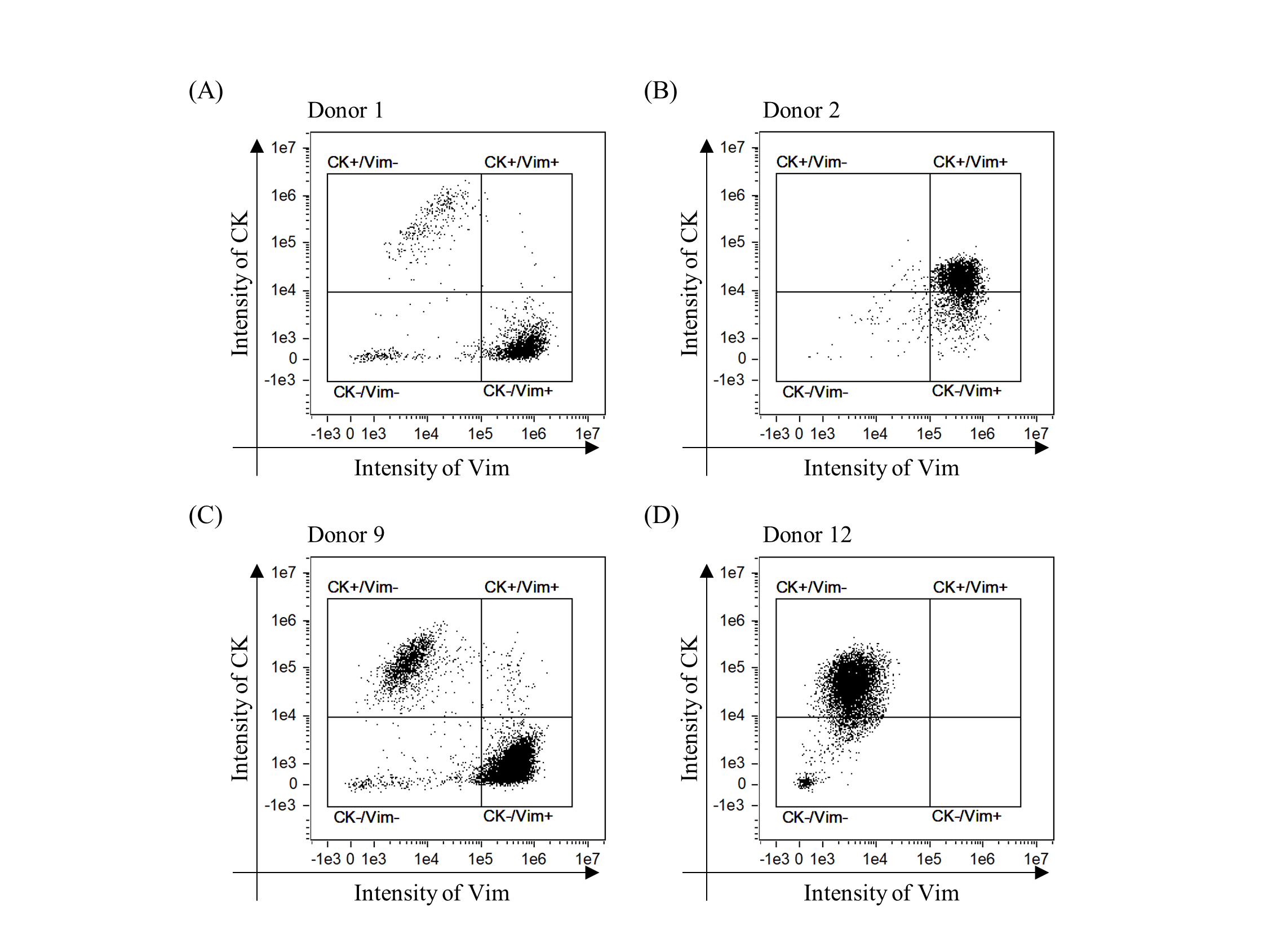

Supplement: Supplementary file 1 [file cancers-17-00303-s001.zip › Figure S2.tif]

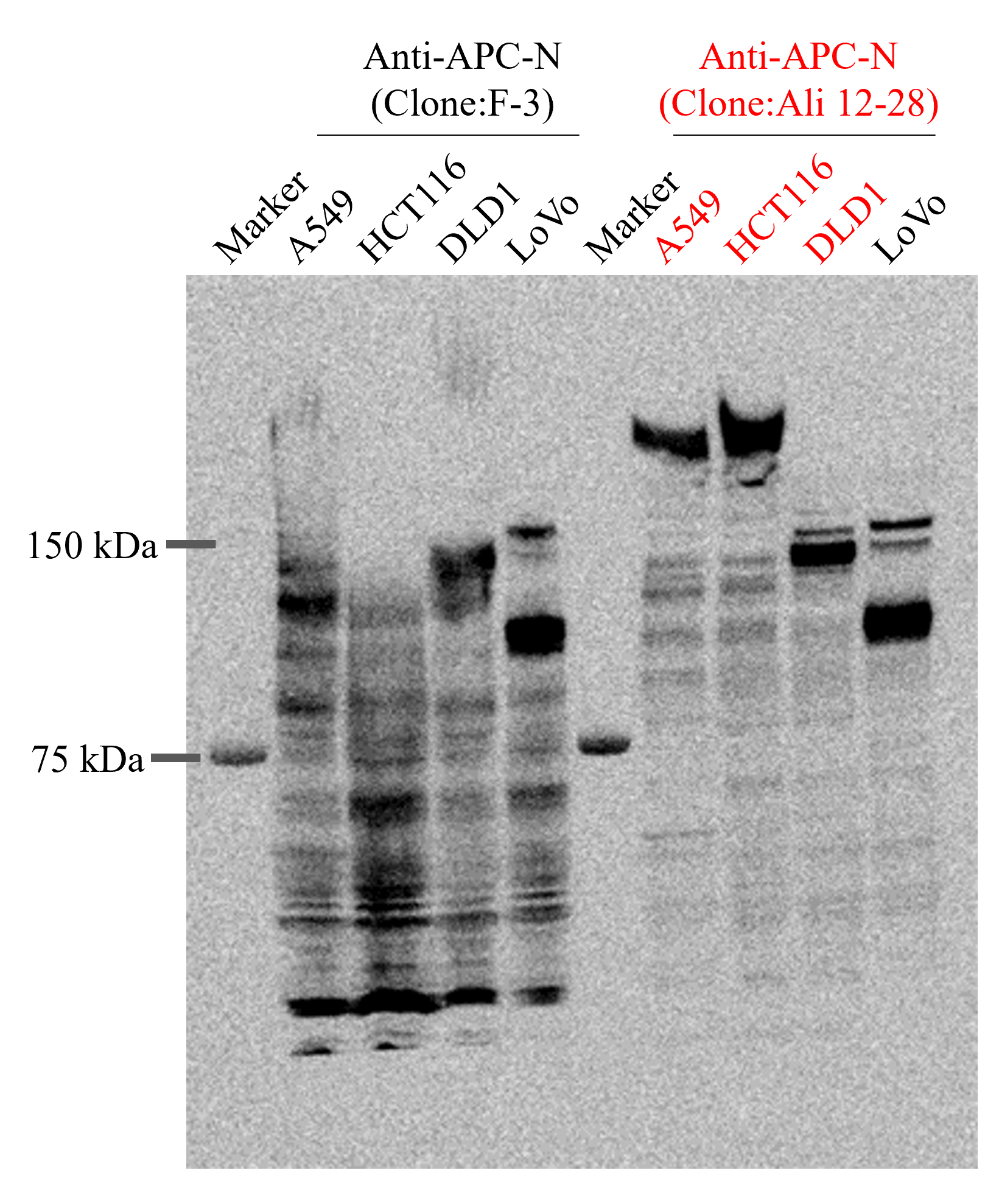

Supplement: Supplementary file 1 [file cancers-17-00303-s001.zip › Figure S3.tif]

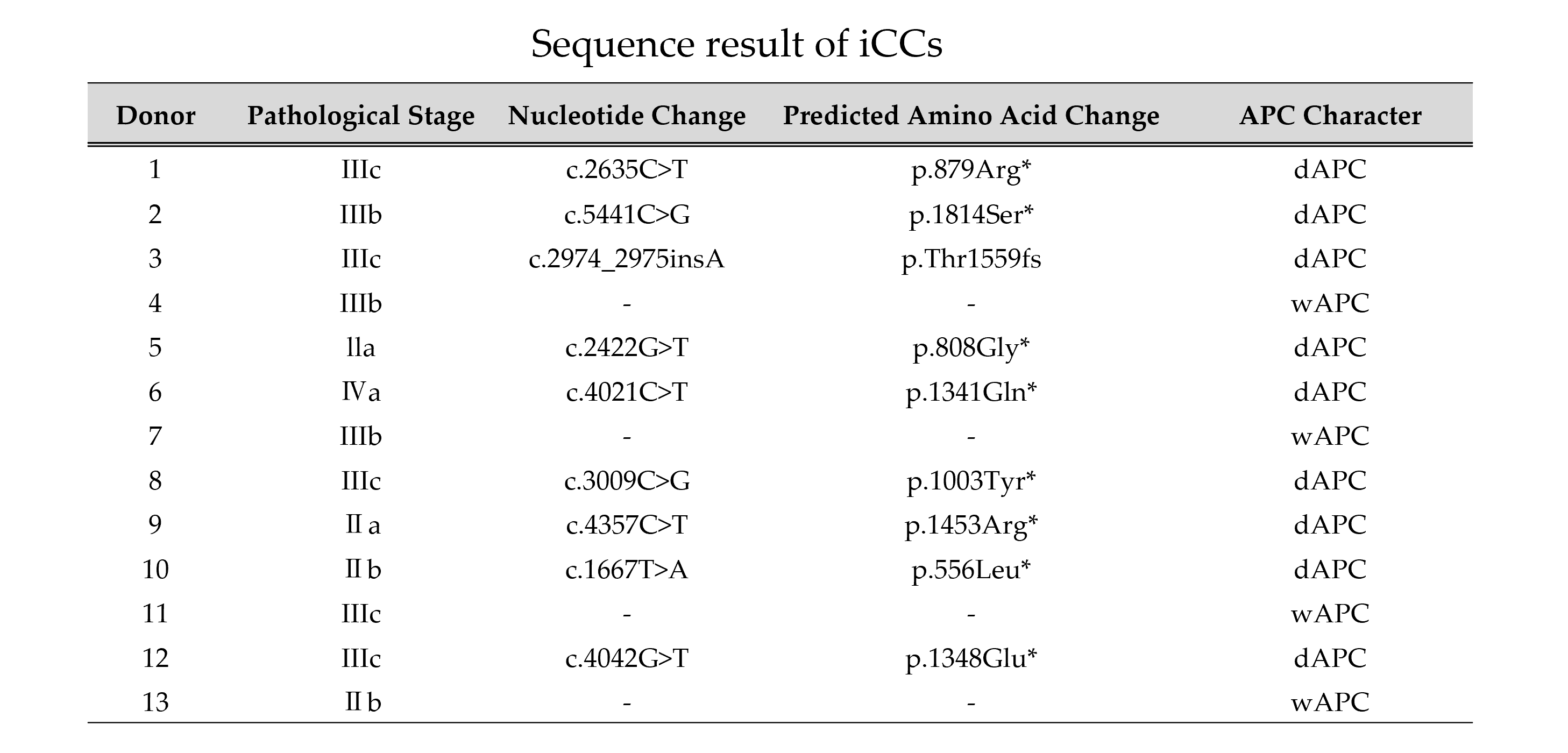

Supplement: Supplementary file 1 [file cancers-17-00303-s001.zip › Table S1.tif]
